# Supplementary material for: Identification of photoperiod-induced specific miRNAs in the adrenal glands of Sunite sheep (Ovis aries)
Source: Front Vet Sci. 2022 Jul 22;9:888207. doi: 10.3389/fvets.2022.888207 (PMC9354845; doi:10.3389/fvets.2022.888207)
Supplement: Supplementary file 1 [file Data_Sheet_1.ZIP › Supplementary file/miRDeep2 file.zip!/miRDeep2 file/Know_Structure/SP42A1/oar-mir-30d.pdf]

miRBase precursor : oar-mir-30d
Total read count : 169677
oar-miR-30d read count : 169137
remaining reads : 540

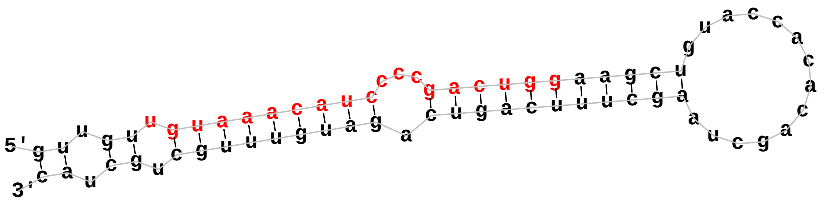

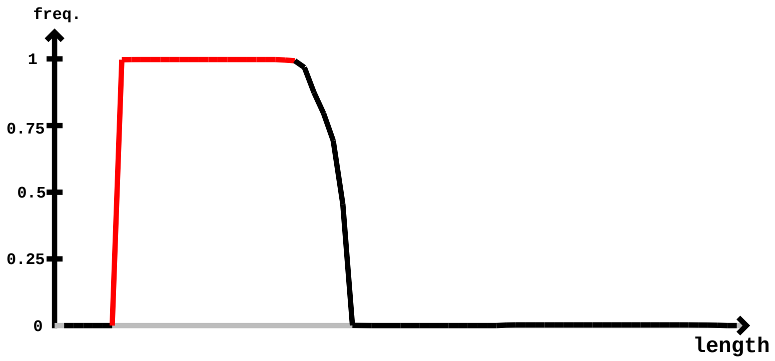


```
      oar-miR-30d
5'- guuguuguaaacauccccgacuggaagcuguaccacacagcuaagcuuucagucagauguuugcugcuac   -3'    exp
    ...((.(((((((((...((((((((((((.............))))))))))).))))))))).))...    reads      mm         sample
    ....uuguaaacauccccgacuggaagc.......................................    1          0          seq
    .....uguaaacaucccUgacu.............................................    4          1          seq
    .....uguaaacauccccgacu.............................................    314        0          seq
    .....uguaaacauccccgacug............................................    458        0          seq
    .....uguaaacauccccgacugg...........................................    4236       0          seq
    .....uguaaacauccccgacugga..........................................    15956      0          seq
    .....uguaaacauccccgacuggaa.........................................    13272      0          seq
    .....uguaaacauccccgacuggaag........................................    17284      0          seq
    .....uguaaacauccccgacuggaagc.......................................    40414      0          seq
    .....uguaaacauccccgacuggaagcu......................................    77070      0          seq
    .....uguaaacauccccgacuggaagcug.....................................    73         0          seq
    .....uguaaacauccccgacuggaagcugu....................................    42         0          seq
    .....uguaaacauccccgacuggaagcugua...................................    1          0          seq
    .....uguaaacauccccgacuggaagcuguacc.................................    1          0          seq
    .....uguaaacauccccgacuggaagcuguacca................................    1          0          seq
    ......guaaacauccccgacugg...........................................    1          0          seq
    ......guaaacauccccgacugga..........................................    6          0          seq
    ......guaaacauccccgacuggaa.........................................    7          0          seq
    ......guaaacauccccgacuggaag........................................    7          0          seq
    ......guaaacauccccgacuggaagc.......................................    31         0          seq
    ......guaaacauccccgacuggaagcu......................................    51         0          seq
    .......uaaacauccccgacugga..........................................    3          0          seq
    .......uaaacauccccgacuggaa.........................................    3          0          seq
    .......uaaacauccccgacuggaag........................................    3          0          seq
    .......uaaacauccccgacuggaagc.......................................    6          0          seq
    .......uaaacauccccgacuggaagcu......................................    10         0          seq
    ...........................................cuuucagucagauguuugc......    13         0          seq
    ...........................................cuuucagucagauguuugcu.....    50         0          seq
    ...........................................cuuucagucagauguuugcug....    31         0          seq
    ...........................................cuuucagucagauguuugcugc...    97         0          seq
    ...........................................cuuucagucagauguuugcugcu..    127        0          seq
    ...........................................cuuucagucagauguuugcugcua.    2          0          seq
    ............................................uuucagucagauguuugc......    1          0          seq
    ............................................uuucagucagauguuugcu.....    3          0          seq
    ............................................uuucagucagauguuugcug....    3          0          seq
```

oar-miR-30d

```
guuguuguaaacauccccgacuggaagcuguaccacacagcuaagcuuucagucagauguuugcugcuac
..............................................uuucagucagauguuugcugc...    18    0    seq
..............................................uuucagucagauguuugcugcu..    77    0    seq
```
